# Childhood asthma phenotypes and endotypes: a glance into the mosaic

**DOI:** 10.1186/s40348-023-00159-1

**Published:** 2023-08-30

**Authors:** Francesco Foppiano, Bianca Schaub

**Affiliations:** 1 Department of Pulmonary and Allergy, Dr. Von Hauner Children’s Hospital, LMU Munich, 80337 Munich, Germany; 2 German Lung Centre (DZL), CPC-Munich, 80337 Munich, Germany

**Keywords:** Childhood asthma, Asthma, Phenotypes, Endotypes

## Abstract

**Background:**

Asthma is an inflammatory lung disease that constitutes the most common noncommunicable chronic disease in childhood. Childhood asthma shows large heterogeneity regarding onset of disease, symptoms, severity, prognosis, and response to therapy.

**Main body:**

Evidence suggests that this variability is due to distinct pathophysiological mechanisms, which has led to an exhaustive research effort to understand and characterize these distinct entities currently designated as “endotypes.” Initially, studies focused on identifying specific groups using clinical variables yielding different “clinical phenotypes.” In addition, the identification of specific patterns based on inflammatory cell counts and cytokine data has resulted in “inflammatory endotypes.” More recently, an increasing number of molecular data from high-throughput technology (“omics” data) have allowed to investigate more complex “molecular endotypes.”

**Conclusion:**

A better definition and comprehension of childhood asthma heterogeneity is key for improving diagnosis and treatment. This review aims at summarizing the current knowledge on this topic and discusses some limitations in their application as well as recommendations for future studies.

## Background

Asthma is the most common inflammatory disease of the airways in childhood being characterized by cough, wheeze, and shortness of breath. Scientific evidence indicates that asthma is a heterogeneous disease encompassing different pathophysiological mechanisms [1]. This has led to the imperative task of identifying and describing the molecular mechanisms underlying this heterogeneity. A better understanding of these mechanisms will result in a more precise definition of asthma, a better characterization of specific risk factors, and a more accurate identification of individuals with poor prognosis [2]. It will also set the grounds for a “personalized” therapy approach, thus alleviating the needs of individuals who do not respond well to therapy and reducing the burden of current therapy side effects [3]. Initially, research efforts to understand this heterogeneity focused on adults due to the medical need of a large population, ethical considerations in children, and sample accessibility [4]. However, it became clear that children with asthma differ in many aspects from their adult counterparts, highlighting the importance of investigating this age group separately [4–6]. This has resulted in many studies aiming at disentangling this heterogeneity using different and novel strategies [7–14]. This review provides a brief overview of different factors that contribute to asthma heterogeneity, defines concepts, and includes respective studies on “phenotypes “ and “endotypes.” Finally, current limitations, open questions, and future perspectives in relation to asthma heterogeneity are discussed.

## Heterogeneity in childhood asthma

### Factors and challenges

Based on differences in the onset and progression of the disease, the severity of symptoms, frequency of exacerbations, inflammatory profiles, and response to therapy, childhood asthma is considered a heterogeneous disease [15]. Early on, physicians recognized this variability, where one example constituted the “extrinsic” and “intrinsic” asthma phenotypes described by Rackeman in 1947 [16]. Since then, our understanding of asthma heterogeneity has increased, leading to the classification of asthma as a syndrome [1, 17].

The heterogeneity in childhood asthma is due to different pathological mechanisms originating from the complex interplay between genetic, epigenetic, and environmental factors [18, 19]. Two recently published reviews describe early-life exposures and their interactions with genetic factors in childhood asthma [19, 20]. These factors can increase the risk of incidence and severity as air pollution exposure or decrease it like growing up on a farm [21, 22]. In addition, the environment can also influence the prevalence of subtypes of asthma. Krautenbacher and colleagues reported that genetics has a strong influence on asthma among farm children, while among non-farm rural children, the environment is the stronger determinant [23].

Genetics also plays an important role in childhood asthma, where family history of asthma remains one of the most important risk factors [24, 25]. Noteworthy is the 17q12-21 locus, which has been replicated several times and is associated with early-onset asthma in childhood [26, 27]. Finally, different epigenetic mechanisms associated with childhood asthma have been identified, where methylation and acetylation are the best described [28]. Also, evidence of the importance of different noncoding RNA populations (e.g., long noncoding (lnc) RNA, micro (mi)RNA) in asthma has started to amount [29].

The inherent challenges of understanding heterogeneity in complex diseases are increased in childhood asthma due to differences in epidemiological definitions and a casual use of asthma severity and control classifications [30]. Van Wonderen and colleagues highlighted this issue by analyzing 122 cohorts and reporting 60 different asthma definitions, which affected incidence, morbidity, and prevalence estimates [31]. In addition, the current definitions of asthma have their limits for a proper diagnosis in preschool children, given that many include objectively measured outcomes that cannot be performed in this age group. This topic has been recently reviewed by Conrad and colleagues [32]. Thus, a more precise and comprehensive definition of asthma addressing these limitations would facilitate our efforts to understand its heterogeneity.

### Description of heterogeneity

**Fig. 1:**
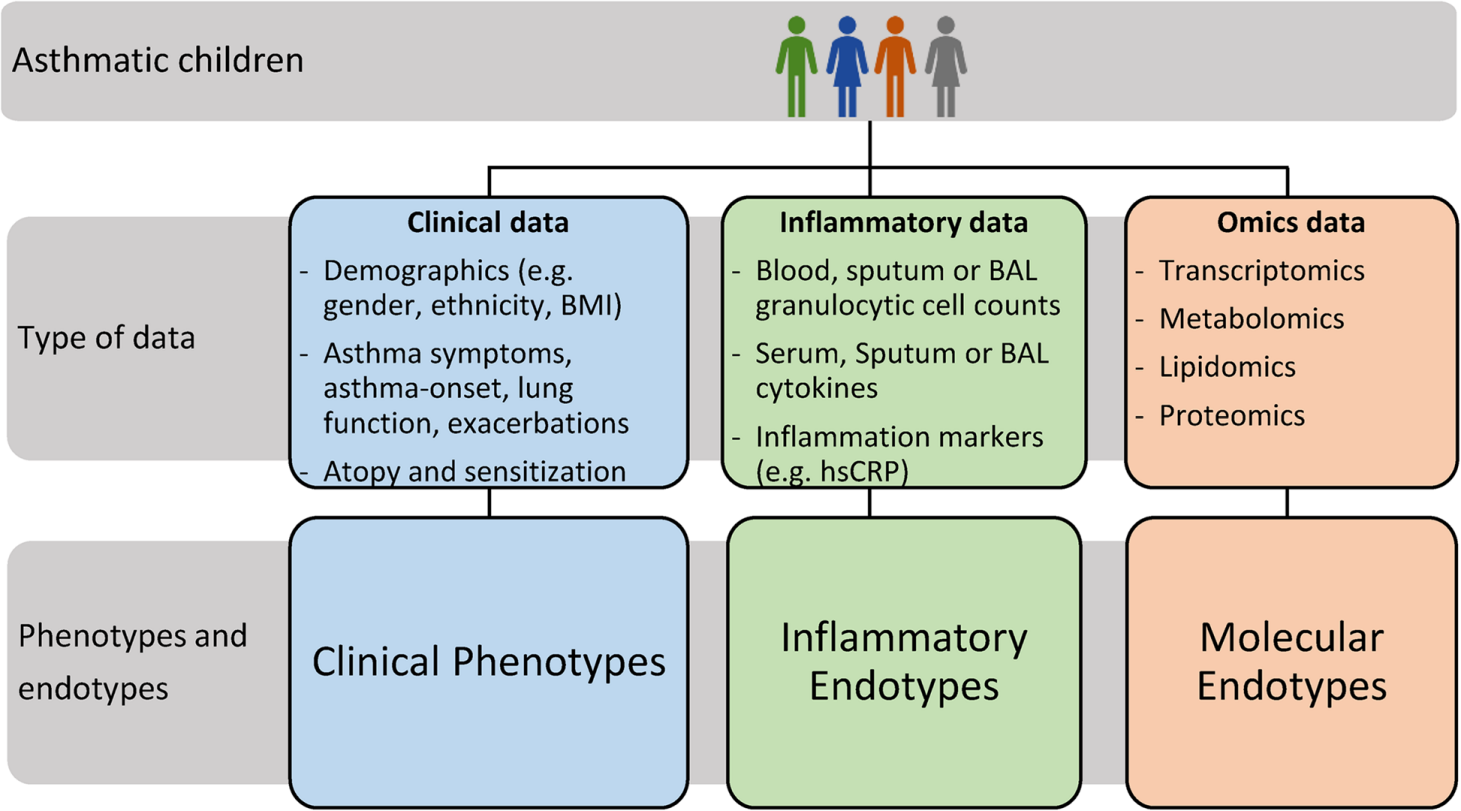
Overview on data used to define different phenotypes and endotypes. Heterogeneous asthmatic children are depicted on the top of the figure. Data type level specifies which kind of data must at least be present to identify specific groups resulting in clinical phenotypes, inflammatory endotypes, and molecular endotypes

Different approaches have been used to disentangle asthma heterogeneity, resulting in multiple phenotypes and endotypes. Phenotypes can be defined as a cluster or group that shares visible or measurable properties like demographic variables, symptom frequency, or physiological properties, whereas an endotype describes a specific biological mechanism which explains the disease [33]. Primarily, two different methods are used for their characterization: (i) hypothesis-driven approaches and (ii) supervised, unsupervised, and model-based clustering methods (data driven). Hypothesis-driven approaches mostly consist of analyzing one or two different traits using clinically relevant cutoffs. On the other hand, supervised and unsupervised clustering methods usually integrate large amount of data using a data-driven approach to identify specific patterns [4]. Depending on the variables used, different phenotypes and endotypes have been identified. These can be broadly classified as clinical phenotypes, inflammatory endotypes, and molecular (“omic” based) endotypes (Fig. 1). The following sections summarize studies describing these phenotypes and endotypes.

## Clinical phenotypes

Clinical phenotypes related to childhood asthma depend on the variables and methods used for identification, as well as the aim of the study. Pijnenburg and colleagues defined four domains where these phenotypes are most useful in clinical practice. These include diagnosis, pathophysiology, prediction, and response to therapy [20]. Many studies have identified clinical phenotypes in preschool wheezing children and school-age asthmatic children [11, 34, 35]. Children defined as preschool wheezer present with pulmonary symptoms such as wheeze with differences in time of onset, frequency, duration, and severity of symptoms. They cannot perform lung function tests due to their young age [32]. Yet, wheezing constitutes one of the most significant risk factors for a diagnosis of childhood asthma [36]. Importantly, the two terms preschool wheezing and childhood asthma are distinct with only partial overlap and thus require thorough definition in order to classify phenotype groups correctly.

### Clinical wheeze and asthma phenotypes

The assessment of triggers and the persistence of wheezing symptoms have resulted in two phenotypes among preschool wheezing children [37]. One is the episodic viral wheeze (EVW) phenotype that is characterized by wheezing symptoms only during viral infections and no symptoms in between. The other one is the multi-trigger wheeze (MTW) phenotype, which presents with wheezing symptoms during viral infections but also in response to different factors like allergens or exercise [37]. The EVW phenotype is more frequent in infancy and tends to become asymptomatic later in life. On the other hand, the MTW phenotype is strongly associated with lung function deficits and is more likely to persist into school age [13, 35]. While a recent study demonstrated the stability of these phenotypes, highlighting their value in the clinical setting, physicians have also scrutinized the usefulness of these phenotypes [13, 38, 39].

Among asthmatic children, two phenotypes have been identified based on the presence of atopy. Allergic asthma is characterized by allergen sensitization (specific IgE) and the presence of atopic diseases (e.g., atopic dermatitis, allergic rhinitis). Nonallergic asthma shows no allergic sensitization nor additional atopic diseases [11, 40]. Allergic asthma is far more common in childhood, being strongly associated with eosinophilic inflammation and type 2 (T2) biomarkers. Most of these children tend to respond well to inhaled corticosteroids (ICS) [41].

### Phenotypes related to prediction

Prediction of asthma diagnosis in children is clinically relevant, and the description of longitudinal patterns of asthma-related traits results in phenotypes with different predictive values for asthma diagnosis. Sensitization is a key feature of allergic asthma, but the mechanisms underlying this relationship remain unclear. Recent studies investigated longitudinal trajectories of this trait and assessed their risk for an asthma diagnosis [9, 42, 43]. One common observation was that infants in early life (< 1 year) were rarely sensitized, indicating little predictive value of sensitization data at this time point. In addition, persistent sensitization to house dust mite was associated with an increased risk of asthma later in life [42, 43]. Hose and colleagues also described a trajectory characterized by high values of specific IgE to multiple allergens in two independent cohorts with an increased risk of developing asthma [9].

The temporal trajectories of wheeze symptoms have also been investigated using hypothesis-driven or latent class analysis (LCA) methods. These studies consistently describe four distinct trajectories (never or infrequent wheeze, early onset wheeze, late-onset wheeze, and recurrent wheeze). In addition, studies using LCA algorithms report a fifth intermediate trajectory. Children following the late onset or the recurrent wheezing trajectories have an increased risk for lung function abnormalities and asthma diagnosis [34, 36, 44].

### Phenotypes related to therapy response

Current treatment guidelines, such as the Global Initiative for Asthma (GINA), are based on the use of ICS; however, specific clinical sub-phenotypes are not included [45]. Although these guidelines have proven effective in controlling asthma symptoms, a subgroup of children are still symptomatic. Two distinct groups are particularly important based on their disease severity: difficult to treat asthma and severe therapy-resistant asthma (STRA). The former describes children who due to modifiable factors (e.g., poor adherence, incorrect use of inhaler) respond poorly to therapy. The latter comprises children who show poor responses to therapy even after addressing these modifiable factors [15]. This observation has prompted the research on differences in therapy response to transition from a “one-size-fits-all” treatment to a more personalized approach [10, 46]. Therapies based on monoclonal antibodies targeting specific pathways implicated in asthma are showing already satisfactory results [47].

Two recent studies classified asthmatic children and adolescents from the Asthma Phenotypes in the Inner City (APIC) study into two groups: “difficult-to-control” (DTC) and “easy-to-control” (ETC) asthma. The DTC group included children requiring high doses of ICS over a 1-year period (6 visits) whereas the ETC children requiring low doses. It is important to mention that this group designation did not reflect asthma control [7, 48]. Key features separating both groups include atopy-related traits, rhinitis severity score, and body mass index (BMI). A reduction of controller requirement after the follow-up period was observed only in the ETC group [48]. In a subsample of African-American children from the same cohort, the same two groups were compared including additionally inflammation and cytokine data. The DTC group presented higher percentage of blood eosinophils and neutrophils at baseline and a positive association with chemokine C-X-C motif ligand (CXCL)-1, interleukin (IL)-17A, IL-8, and IL-5, while IL-4 and IL-13 were positively associated with the ETC group [7]. This is interesting because it associates the DTC group with a mixed inflammatory profile (explained in the inflammatory section), while ETC group shows a T2 dominant inflammatory profile.

### Phenotypes identified by clustering algorithms

In this section, different studies using clustering methods on specific children populations (e.g., severe asthmatic children or preschool wheezing) are described, which aimed at identifying sub-phenotypes within these groups. When investigating severe asthmatic children, four distinct clusters were identified in a sample from the Severe Asthma Research Program (SARP) using a data-driven approach [8]. Three clusters included children with early onset asthma and differing levels of atopy, T2 inflammation markers, and airflow obstruction. The fourth cluster included children with late-onset asthma, low atopy levels, and normal lung function [8]. Schatz and colleagues used a similar approach in an asthmatic children sample and identified five clusters, which differed in gender, ethnicity, lung function, and atopic burden [49].

Clustering methods have also been used to investigate a diverse school-aged asthmatic children population with differing severity and atopy levels. Zoratti and colleagues identified five distinct clusters in asthmatic children from the APIC study that covered all possible combinations of atopy and symptom severity [14]. Three clusters showed increasing levels of atopy and an increasing correlation with blood eosinophils, while the remaining two clusters had low levels of atopy. Noteworthy is the cluster designated as “cluster B,” which had the lowest atopy level, although it included many children requiring high doses of asthma controller therapy.

Another study took a similar approach and used these methods on recurrent wheezing children from the Childhood Asthma Prevention Study (CAPS) [12]. These clusters were classified as atopic wheezers, nonatopic wheezers, transient wheezers, paradoxical responders, and remitters. The atopic and nonatopic wheezers were the most severe groups showing similar American Thoracic Symptom report (ATS-B) scores and lung dysfunction but differing significantly in atopy markers. Notably, these clinical parameters remained stable in these two groups at the 7-year follow-up. In addition, the follow-up included the measurement of fractional exhaled nitric oxide (FeNO), which was significantly higher in the atopic wheezer group. Interestingly, cluster “B” from the APIC study is similar to the nonatopic wheezers described in the CAPS study. In all these studies, an atopic and a nonatopic cluster were identified; additionally, these groups could be separated in distinct subgroups with specific characteristics associated with differences in severity and exacerbation history.

These unbiased methods have proven to be relevant for therapy response, where a differential response to treatment has been observed between different clusters. Howrylak and colleagues applied clustering methods in asthmatic children from the Childhood Asthma Management (CAMP) trial, a three-arm-randomized clinical trial comparing the treatment with budesonide, nedocromil, and placebo. Cluster identification was based on atopic burden, lung function, and exacerbation history [10]. The original study showed that only budesonide had a significant reduction in asthma exacerbations and additional use of asthma controller medicine compared to placebo. When reanalyzing the data stratified by clusters, three of the five clusters showed a similar trend. However, children in a low atopic cluster with high exacerbations had a beneficial response to both treatments compared to placebo, whereas children in the severe atopic cluster responded poorly to budesonide and nedocromil [10]. Chang et al. also reported differences in therapy across clusters when using clustering analysis on data from another asthma treatment clinical trial [46].

Finally, asthma phenotypes have been almost exclusively described in cohorts from European countries and the USA. However, due to the increase in asthma incidence in South America, Asia, and Africa, studies identifying asthma phenotypes from these regions are emerging [50–53]. One example is the study by Yoon and colleagues who performed a clustering analysis in school-aged asthmatic children from the Korean childhood Asthma Study (KAS), identifying four distinct clinical phenotypes. Similar to previous reports, most clusters (three) were atopic, while one was non-atopic. Notably, two of the atopic phenotypes included predominantly male children; these phenotypes were also characterized by early-onset asthma compared to the female predominant cluster that included mostly children with late-onset asthma [54]. Another study analyzed wheezing trajectories in South-African preschool-aged children. Even though the trajectories were similar to those described in European cohorts, differences regarding the age of onset and duration of symptoms between “equivalent” trajectories were found [55]. The characterization of phenotypes in different populations will help understand the impact of the environment and the genetic background on asthma heterogeneity.

To sum up, many different clinical phenotypes have been identified, some of which are currently relevant to clinical practice like the STRA phenotype. In addition, many of the clinical phenotypes that were originally identified using hypothesis-driven methods are being confirmed and expanded by clustering analysis (e.g., allergic and nonallergic asthma) (Table 1). However, clinical phenotypes need to be better characterized to avoid overlap, which will make them more useful for early identification of children at high risk of developing asthma or improve treatment assignment.

**Table 1:** Overview of clinical phenotypes in asthmatic children associated with cluster analyses and inflammatory endotypes

**Analyzed asthmapopulation**	**Clinical phenotypes using hypothesis-driven methods**	**Clinical phenotypes based on clustering methods**	**Inflammatory endotypes**
Severe asthmatics (poor response to ICS)	Severe treatment-resistant asthma (STRA): Poor ICS response independent of modifiable factors [15]	SARP cohort: Four clusters (three atopic, one non-atopic) with differences in asthma onset and lung function [8] TENOR cohort: Five clusters (four atopic, one non-atopic) with differences in gender, ethnicity, and lung function [49]	T2-high (eosinophilic) [56] Eosinophilic non-T2-high [57, 58] Neutrophilic [59, 60] Paucigranulocytic [61] Mixed granulocytic [62]
	Difficult-to-treat asthma: Poor response to ICS due to modifiable factors [15]		
Mild, intermediate, and severe asthmatics	Allergic asthma: Presence of atopy and T2 biomarkers (e.g., FeNO) [40]	APIC cohort: Five clusters (three atopic and two non-atopic) with differences in lung function [14] CAMP trial: Five clusters (three atopic and two non-atopic) with differences in exacerbation rates, lung function, and response to treatment [10] KAS study: Four clusters (three atopic and one non-atopic) [54]	T2-high (eosinophilic) [63–65] Only eosinophilic (based on data from blood) [63] Paucigranulocytic (based on data from blood) [63]
	Nonallergic asthma: Not associated with atopy [11]		

## Inflammatory endotypes

Studies aiming at identifying different inflammatory endotypes among asthmatic children use similar approaches as described before using data of cell counts from different tissues (e.g., bronchoalveolar lavage (BAL), sputum, biopsies, and serum), cytokine measurements, and specific inflammation biomarkers (e.g., FeNO) [4]. Evidence supports the existence of four inflammatory endotypes in asthmatic children, designated as follows: T2-high (eosinophilic), neutrophilic, pauci-granulocytic, and mixed granulocytic asthma [5, 57, 61, 62, 66] (Table 1). A better description of these endotypes may result in the identification of potential new targets for therapy; this is especially relevant for STRA children.

### Inflammatory endotypes identified by hypothesis-driven methods

The most common and best-described endotype is the T2-high (eosinophilic), which is also present in the adult population. This endotype has been described in children with mild, moderate, and severe asthma and is characterized by a high degree of atopy, increased eosinophils (in sputum and serum), high levels of T2 cytokines (IL-4, IL-5, and IL-13), and early signs of airway remodeling [63, 64, 66]. Children with this endotype tend to respond well to ICS; however, a subset was described that did not [56]. Studies in adults suggest that an equivalent endotype should be divided into sub-endotypes, and similar evidence is starting to accumulate in children [67]. Bossley and colleagues compared children with STRA against healthy subjects and observed that the former displayed higher BAL and biopsy eosinophil counts. However, no difference between the groups in the concentration of T2 cytokines in BAL and submucosal IL-5^+^ and IL-13^+^ cells was observed [57]. Children with similar characteristics have also been described in a study assessing therapy response to omalizumab [58]. Thus, this sub-endotype presents a high count of eosinophils but low atopy markers (e.g., IgE levels). In adults, the innate lymphoid cells type 2 (ILC2) has been implicated in the pathological mechanism underlying a similar sub-endotype, yet the role of this cell population remains to be investigated in children [15, 56]. Two phenotypes sharing features with the nonatopic and atopic eosinophilic phenotypes have been described in the German multicenter All Age Asthma Cohort (ALLIANCE), which includes preschool wheezers, school-age asthmatic children, and adult asthmatics [63]. The three populations were grouped separately into four different inflammatory endotypes using clinically relevant cutoffs of blood eosinophilia and atopy levels. These endotypes were consistent in all three age groups and described as eosinophilic atopic (T2-high), only eosinophilic, and only atopic and non-atopic nor eosinophilic (T2-low) [63].

The neutrophilic inflammatory endotype has been identified in children with STRA and is characterized by neutrophilic infiltration of the airways, where a Th1 or/and Th17 skewed immune profile has been implicated [68]. Andersson and colleagues compared children with STRA against healthy controls identifying higher levels of BAL eosinophils, submucosal eosinophils, and intraepithelial neutrophils in endobronchial biopsies of STRA children. These STRA children were divided based on the number of intraepithelial neutrophils, where the group with high neutrophils had better asthma control test (ACT) scores, better lung function, and lower doses of ICS [60]. These results suggest a beneficial role of neutrophil infiltration. However, another study reported two similar groups among STRA children based on BAL neutrophil percentages. They stimulated primary neutrophils from healthy patients with BAL from both groups. Neutrophils stimulated with BAL from the high-neutrophil group had greater phagocytic capacities; increased neutrophil extracellular traps (NET) formation, inflammatory mediators, and granule release; and enhanced survival. All these features point to a detrimental role of neutrophils [59]. Raedler and colleagues also identified among nonallergic asthmatic children a similar immune profile to that seen in neutrophilic asthma. The authors reported a shift towards a Th-17 response and elevated blood neutrophil counts. The more infrequent, mixed granulocytic and the paucigranulocytic endotypes are less well understood and have also been identified in STRA children, where the former is characterized by the presence of neutrophilic and eosinophilic inflammation and the latter by the absence of both inflammatory cells [61, 62].

The non-T2 endotype (neutrophilic and paucigranulocytic) is discussed based on the current unclarity of (i) a novel mechanism underlying these specifics or (ii) just a steroid-driven result of immunosuppression. Solutions to this issue are difficult as characterization via inflammatory cells in the airways or sputum is primarily performed in severe asthmatics, generally requiring high doses of ICS. Importantly, a murine asthma model with low T2 cytokines, neutrophilic inflammation, and elevated levels of IL-17 and interferon (IFN)-γ with bronchial hyperresponsiveness has been developed and may help to elucidate its relevance [69]. In summary, different inflammatory endotypes have been described, and their detailed characterization may direct research towards new therapeutics and contribute to an adequate evidence-based selection of the best treatment for specific groups of asthmatic children.

### Inflammatory endotypes identified by clustering methods

Recent studies are applying clustering methods to identify inflammatory endotypes using cell counts, cytokine data, and clinical parameters. Guiddir and colleagues performed a cluster analysis using clinical variables (demographics, atopy-related variables, severity variables) and inflammatory cell counts (blood and BAL neutrophil and eosinophil counts) in children with recurrent wheeze (preschool age) or severe asthma from the Severe Asthma Molecular Phenotype (SAMP) study [65]. They identified three distinct clusters, one including children with steroid refractory recurrent wheeze (94%) with high counts of blood neutrophils and another consisting of steroid refractory severe asthma with high counts of blood eosinophils. The third cluster included children with controlled recurrent wheeze having intermediate blood cell counts and a high proportion of children with controlled asthma [65]. Another study applied a clustering algorithm using eight variables covering inflammation, infection, sensitization, and ICS usage in preschool-age children with recurrent severe and with non-wheezing respiratory diseases [70]. Blood eosinophils were the only leukocytes that differed between both groups, with higher counts in children with recurrent wheeze. After clustering analysis, four distinct groups were identified, one atopic group with the highest BAL eosinophils cell counts and three non-atopic. Among the non-atopic, one cluster included almost all controls with a similar number of recurrent wheezers who did not receive ICS therapy. Another non-atopic cluster presented the highest counts of blood neutrophils [70]. Salvermoser and colleagues applied a different strategy using a clustering algorithm on cytokine data and characterizing the clusters using specific IgE measurements and clinical variables [71]. Three distinct clusters were identified, and one presented a predominance of IFN-γ/IL-17/IL-5 that agrees with the mixed granulocytic endotype. Here, airway inflammatory cell counts were not measured. This finding is also interesting because this study included children with mild to moderate asthma [71]. As with clinical phenotypes, clustering methods are proven to be useful for a more detailed understanding of inflammatory endotypes.

## Molecular endotypes

Recent technological advances and lowering costs of high-throughput technologies from the “omics” fields are enabling asthma researchers to gather high amounts of molecular data from a large number of individuals [72, 73]. This section will focus on studies using novel approaches combining omics data with clustering methods. These strategies can be used to uncover specific pathophysiological mechanisms underlying the different endotypes [2].

### Transcriptomics-based endotypes

Transcriptomics constitutes the study of a defined RNA population from a cell or tissue that can comprise the whole transcriptome or a subset (e.g., mRNA, miRNA). This field has been intensively studied in relation to asthma in adults and children, reviewed elsewhere [72]. However, studies analyzing this data using clustering algorithms are still sparse and much more common in adults [74, 75]. One recent study identified three clusters using microarray technology in peripheral blood mononuclear cells (PBMCs) from mild to severe asthmatic children and adolescents [76]. One cluster had significantly higher blood eosinophils and ACT scores in combination with the highest levels of FeNO and total IgE and the lowest count of blood neutrophils. A second cluster presented significantly higher levels of blood neutrophils and lower ACT scores; it also had the lowest eosinophil counts and total IgE levels. The third cluster had intermediate values for all variables. These three clusters agree well with the characteristic features of the T2-high (eosinophilic), neutrophilic, and mixed granulocytic inflammatory endotypes, respectively. Notably, the “high neutrophilic” cluster was considered the most severe cluster, and its expression profile was strongly associated with glucocorticoid signaling and activation of Th1/Th17 immune pathways [76]. Another study used data from the same cohort and identified five clusters using 12 clinical variables including blood counts, total IgE levels, and lung function parameters. Expression profiles of subsamples from each cluster were determined using microarray data and compared. Two of the five clusters were predominantly eosinophilic (one non-atopic), while another one was predominantly neutrophilic. Similar to the previously described study, the neutrophilic dominant cluster showed a differentiated gene expression profile compared to the other clusters, where identified genes were associated with corticosteroid response. Transcriptomics plays an essential role early on in asthma pathogenesis, where an early dysregulation in the transcriptome in two year old children preceding asthma manifestation has been found [77]. Thus, more studies identifying specific gene expression profiles among asthmatic children are necessary to elucidate the molecular mechanisms underlying the disease.

### Mass spectrometry-based endotypes

Another fast-evolving field is mass spectrometry (MS) omics, where a defined set of biomolecules (lipids, proteins, or metabolites) are quantified in a cell or tissue. Many studies have assessed the association between these molecules and asthma in children and adults, reviewed elsewhere [78]. However, studies using clustering analysis in asthmatic patients combined with MS-omics data are just starting to evolve. Kelly and colleagues used metabolome-wide data and clustering analysis to identify five clusters in asthmatic children and adolescents from the genetics of asthma in Costa Rica study (GARCS). They found differences in lung function parameters between the clusters and validated these differences in an independent cohort. Different lipid molecules were key features for cluster differentiation suggesting a role of pulmonary surfactant homeostasis in asthma severity [79]. Metabolomics data of exhaled breath condensate have also been analyzed using clustering algorithms, where three identified cluster differed in blood eosinophil percentage, gender, and exacerbation ratio. Hydroxybutyrate and formate were important metabolites for cluster assignment [80].

Infant bronchiolitis is a risk factor for asthma diagnosis. Recent studies use different MS omics and clustering algorithms to identify endotypes in children suffering from acute bronchiolitis. These clusters were characterized, and their risk for developing asthma and recurrent wheeze was prospectively evaluated. Fujiogi and colleagues used lipidomics data from nasopharyngeal swabs and identified four endotypes, one characterized by high atopy and low levels of sphingolipids. Children in this cluster had the highest risk of developing recurrent wheeze and asthma. Interestingly, sphingolipid biosynthesis is regulated by the 17q21 locus gene *ORMDL3* which has been associated with childhood asthma [81]. These results agree with a similar study conducted on the same study sample using metabolomics data [82]. In addition, proteomics data from serum samples of children from this cohort have been analyzed using clustering algorithms, where three endotypes were identified. Children assigned to an endotype characterized by atopy and upregulation of nuclear factor k-light-chain-enhancer (NF-κB) and phosphoinositide-3-kinase (PI3K) signaling pathways were also at higher risk of having an asthma diagnosis at 6 years of age [83].

## Implications and future perspectives

### Importance of phenotypes and endotypes in childhood asthma

In summary, different phenotypes and endotypes have been described in asthmatic children and adults with potential use in clinical practice (diagnosis, prediction, therapy) [20]. These studies have contributed to our better understanding of asthma, and some are starting to shape asthma management, specifically in adults. Notably, sputum eosinophils and FeNO have been included in GINA management guidelines 2022 as alternative strategies for adjustment of asthma treatment in adults and individuals older than 12 years, respectively [45]. In addition, the described inflammatory endotypes provided the theoretical basis for the development of therapeutics based on monoclonal antibodies (biologicals) targeting up- and downstream effectors of asthma-associated signaling pathways. These are mainly directed at T2-high effectors (IgE, IL-5, IL-4); however, biologicals targeting potential T2-low effectors (e.g., neutrophilic asthma) are currently being tested (e.g., IL-1β, IL-17A) [47, 84]. At present, six different biologicals have been approved for their use in children (five anti-T2-high effectors and anti-TSLP), although information on their long-term use in this population is still lacking and is often extrapolated from adults [85]. In addition, none of the biologicals has been approved for asthma in children younger than 6 years of age; thus, clinical trials looking at safety and effectiveness in this population are urgently needed.

### Limitations of asthma phenotypes and endotypes

Although these phenotypes and endotypes have proven valuable for our understanding of asthma, several limitations hinder their inclusion into clinical practice yet, especially in children. One significant limitation is our poor understanding of their temporal stability. Most studies focusing on this topic use a cross-sectional design, thus lacking the assessment of longitudinal features. Many experts have criticized this, which led to the European Respiratory Society task force recommending in 2014 not to treat MTW and EVW phenotypes differently [86]. Researchers also report low stability among inflammatory endotypes [87]. In addition, most of these are described in STRA children where the influence of high ICS doses is unclear [68]. A better understanding of the temporal stability of these phenotypes and endotypes is critical for childhood asthma, which shows higher variability and different trajectories than adult asthma [88]. Thus, researchers aiming at identifying reliable asthma phenotypes and endotypes in children need to implement longitudinal study designs to evaluate them at different time points, yet certainly challenging in practice. Spycher and colleagues recently addressed this issue by estimating transition probabilities between MTW, EVW, and non-wheezers during their first 6 years of life in a sample of around 10,000 children [13]. They replicated their observations in an independent cohort and showed that even though a high proportion of MTW and EVW children become asymptomatic at 6 years, it is more likely that they remain within their phenotypes [13].

Validation and replication of phenotypes and endotypes are also important, specifically for studies using clustering methods. These studies require inclusion of validation sets. Although many report cluster stability measures, only a few conducted proper validation [71]. Of note, an increasing number of studies have identified novel phenotypes and endotypes, but also replication of existing ones is key for confirmation of possible and consistent mechanisms allowing further elucidation of their role as future biomarkers or even therapy options. Chang and colleagues demonstrated in their study that this is feasible by replicating the clusters identified in the SARP cohort by means of careful and thorough statistical methods [46]. Another aspect that hinders replication and comparability between phenotypes and endotypes from different studies is the selection of variables used to create and describe the clusters [32]. Variable selection is a critical step for phenotype and endotype identification. Deliu and colleagues demonstrated this, by testing three different approaches for variable selection. Each method resulted in different phenotypes with poor overlap and different clinical characteristics. The most stable phenotypes were selected using a blended approach combining clinical experts and appropriate biostatistical methods [53].

### Recommendations for future clustering analyses

Considering the high numbers of current studies using clustering methods, developing a guideline for these studies would help coordinate efforts across research groups. An interdisciplinary-based expert guideline aiming to include a set of standardized variables for cluster characterization, detailed clinical characterization, and study proceeding with carefully planned statistical strategies would greatly facilitate across-study comparisons and enable meta-analyses. Additional variables can still be included and presented as sensitivity analyses. Conrad and colleagues have proposed relevant features for this [32]. Finally, the study by Fujiogi and colleagues shows impressively how different data (clinical data, viral infection data, lipidomics data) can first be clustered separately and in a second step combined [81]. Research in this direction will bring us closer to a systems biology approach, which analyzes different data “types” in an integrative manner.

## Conclusions

Several phenotypes and endotypes have emerged for childhood asthma, reflecting variability in clinical parameters, inflammatory profiles, and molecular mechanisms. Information regarding their temporal stability and reproducibility is lacking and can provide important addition for future studies. Recent advances in statistical methods and molecular techniques are currently applied, and more comprehensive analyses following a systems biology approach are expected. The identification of stable phenotypes and endotypes will change our understanding and the management of childhood asthma. This goal will be achieved by a careful combination of omics data with immunological functional assessment.

## Data Availability

Not applicable.

## Abbreviations

lncRNA: Long-noncoding RNA
miRNA: MicroRNA
EVW: Episodic viral wheeze
MTW: Multi-trigger wheeze
T2: Type 2
ICS: Inhaled corticosteroid
IgE: Immunoglobulin E
LCA: Latent class analysis
GINA: Global Initiative for Asthma
STRA: Severe therapy-resistant asthma
DTC: Difficult to control
ETC: Easy to control
APIC: Asthma Phenotypes in the Inner city
BMI: Body mass index
CXCL: Chemokine C-X-C motif ligand
IL: Interleukin
CAPS: Childhood Asthma Prevention Study
KAS: Korean childhood Asthma Study
ATS-B: American Thoracic Society symptom report
FeNO: Fractional exhaled nitrogen oxide
BAL: Bronchoalveolar lavage
ILC2: Innate lymphoid cell type 2
ALLIANCE: All Age Asthma Cohort
SAMP: Severe asthma molecular phenotype
IFN: Interferon
PBMC: Peripheral blood mononuclear cells
MS: Mass spectrometry
ACT: Asthma control test
GARCS: Genetics of asthma in Costa Rica study
TSLP: Thymic stromal lymphopoietin
NFκB: Nuclear factor k-light-chain-enhancer
PI3K: Phosphoinositide-3-kinase
